# Structural Inequalities in Online Health Information Seeking: Cross-National Multilevel Study

**DOI:** 10.2196/88110

**Published:** 2026-05-19

**Authors:** Petra Raudenská, Elena Link

**Affiliations:** 1 Institute of Sociology, Czech Academy of Sciences, Jilská 1, Prague, 11000, Czech Republic, 420 210 310 238; 2 Department of Communication, Johannes Gutenberg-University Mainz, Jakob-Welder-Weg 12, Mainz, Germany

**Keywords:** online health information seeking, digital health, digital divide, multilevel analysis, cross-national comparison, socioeconomic development, structural determinants, health inequalities, ISSP, International Social Survey Programme

## Abstract

**Background:**

Online health information–seeking behavior (OHISB) has become an increasingly common component of contemporary health self-management. Individuals use a wide range of digital sources, including websites, social media platforms, and mobile apps, to obtain health-related information. However, substantial disparities persist in who seeks health information online, and which populations benefit from digital health resources. While previous research has largely focused on individual-level determinants, cross-national evidence on structural influences remains limited.

**Objective:**

This study aims to assess between-country variation in OHISB, examine associations between individual-level characteristics and OHISB, and investigate how country-level structural conditions are associated with cross-national differences in OHISB, net of individual-level characteristics.

**Methods:**

Data were drawn from the Health and Health Care II module of the International Social Survey Programme (ISSP 2021‐2024; n=35,592; 32 countries). OHISB was measured as any use of the Internet to search for health-related information during the past 12 months. Multilevel logistic regression models were estimated. Country-level indicators were reduced using principal component analysis into 4 composite indices. Robustness checks included analyses excluding respondents without internet access and models incorporating survey weights.

**Results:**

OHISB varied substantially across countries (intraclass correlation coefficient=0.177). At the individual level, younger age, higher education, female respondents, recent health problems, doctor visits, unmet medical needs, and perceived usefulness of the internet were associated with higher odds of OHISB. At the macro level, the socioeconomic and health development showed the strongest association (odds ratio=1.52 per SD; *P*=.003) and explained a substantial share of between-country variation. Cultural hierarchy–individualism was associated with OHISB in separate models but attenuated when adjusted for development. Cross-level interactions indicated that the gender gap and the role of perceived usefulness were more pronounced in higher-development contexts, although these findings were exploratory.

**Conclusions:**

OHISB is associated with both individual characteristics and broader structural conditions. Socioeconomic and health development appear to play a key contextual role in shaping cross-national differences in digital health engagement, highlighting the importance of addressing both individual and structural dimensions of digital health inequalities.

## Introduction

### Background

The rapid expansion of digital health information has transformed how individuals access and manage health-related knowledge. Today, individuals obtain health information from a wide range of online sources, including search engines, institutional and news websites, mobile apps, online forums, social media platforms, or video-based content, and increasingly also artificial intelligence (AI)-assisted search tools and conversational interfaces [1,2]. These resources provide access to diverse types of health content, ranging from preventive advice and lifestyle guidance to information on symptoms, medical treatments, and health care services, which individuals use to understand symptoms, evaluate treatment options, manage chronic conditions, regulate emotions, and support health-related decision-making [3-6]. While digital technologies may enhance access to information and support more active participation in health-related decision-making [7,8], they also introduce new challenges, including information overload, misinformation, and inequalities in individuals’ ability to access and benefit from online health information [9,10].

### Social Stratification and Online Health Information Seeking

These disparities reflect broader patterns of social and digital inequality. Stratification theory suggests that existing social hierarchies tend to be reproduced in digital environments rather than eliminated by technological access alone [11-13]. Accordingly, the benefits of digital technologies are unevenly distributed across population groups. Engagement in online health information seeking varies with socioeconomic resources, education, age, digital skills, health literacy, and trust in online information sources, reflecting both structural and motivational barriers to online engagement. Non-engagement may therefore result either from lack of internet access or from active disengagement driven by low perceived utility, distrust, or preferences for alternative sources [14]. Moreover, the types of digital platforms and information sources used for health information seeking also differ across demographic and cultural groups, reflecting variations in media preferences, language constraints, and health information practices [15,16].

From this perspective, online health information–seeking behavior (OHISB) can be understood as a behavioral manifestation of digital stratification. Empirical research shows that individuals with greater socioeconomic resources, education, and digital skills are more likely to access and benefit from online health information, whereas disadvantaged groups face persistent barriers to engagement. Women, younger individuals, and those with higher levels of education or income tend to seek health information online more frequently, whereas older adults, individuals with lower socioeconomic status, rural residents, and racial minorities are less likely to engage in such behaviors [4,17-19].

In addition to structural resources, health-related circumstances and psycho-motivational factors also shape online health information seeking. Individuals experiencing health problems, perceived health risks, or higher levels of health anxiety are more likely to search for health information online [20,21]. Information-seeking models further highlight the importance of perceived usefulness and credibility of information sources, knowledge gaps, and self-efficacy in shaping engagement with online health information [22-24].

### From Individual Determinants to Cross-National Contexts

Inequalities in OHISB extend beyond individuals and are also reflected in substantial differences between countries. In high-income countries, between 70% and 80% of adults report searching for health information online, compared to substantially lower rates in many lower-income settings [4,25,26]. Even within Europe, prevalence ranges from approximately 43% in Bulgaria to more than 80% in Finland [27].

These patterns suggest that engagement with online health information is associated not only with individual characteristics but also with broader structural conditions [28-30]. National differences in health care systems, digital infrastructure, public health conditions, and socioeconomic development can affect both the availability of online health resources and individuals’ capacity to benefit from them [31-34]. For example, constraints in health care access, such as limited availability of medical services, high costs, or long waiting times, may encourage individuals to seek complementary information online, whereas well-functioning health care systems with high levels of trust and satisfaction may reduce this need [35].

Cultural contexts may further shape norms of health-related help-seeking, trust in institutions, and attitudes toward autonomy in decision-making. Societies characterized by higher individualism and lower power distance may encourage more autonomous health information seeking and greater willingness to verify or supplement medical advice through independent sources, whereas more hierarchical or collectivist contexts may rely more strongly on interpersonal networks or professional guidance [36-39].

Digital infrastructure defines the technological opportunity structure for online engagement. Countries with higher levels of internet access, connectivity, and digital adoption provide individuals with greater opportunities to access and use online health information, while differences in digital skills and information literacy influence individuals’ ability to navigate online information environments and evaluate the credibility of health content [40-42].

Finally, broader socioeconomic development forms the structural foundation for digital engagement and health information access. Higher levels of development are associated with better health care infrastructure, higher educational attainment, stronger digital skills, and greater digital connectivity, all of which facilitate engagement with online health information [42-44]. Conversely, lower levels of development are often linked to weaker infrastructure, lower health literacy, and limited trust in digital sources, potentially constraining engagement with online health information. Regulatory and institutional contexts may further shape the online health information environment by influencing the credibility, visibility, and governance of digital health content across platforms [45-47].

Despite growing recognition of these disparities, research on OHISB remains fragmented. Existing studies have primarily focused on individual-level determinants, while the role of macro-level contexts has received comparatively less systematic attention in large-scale cross-national analyses [9,48]. Moreover, many studies rely on descriptive comparisons or analyses based on a limited number of countries, often without applying multilevel analytical frameworks, making it difficult to disentangle the relative contributions of individual and contextual factors. As a result, the relative contribution of individual and contextual factors to cross-national variation in OHISB remains insufficiently understood.

### Study Objectives

This study addresses this gap by examining cross-national variation in OHISB using a comparative multilevel framework. The objectives of this study are threefold: (1) to assess between-country variation in OHISB, (2) to examine associations between individual-level characteristics and OHISB, and (3) to investigate how country-level structural conditions are associated with cross-national differences in OHISB. The analysis draws on data from the Health and Health Care II module of the International Social Survey Programme (ISSP 2021-2024; 32 countries; n=35,592), a long-running cross-national survey program that collects harmonized social science data using probabilistic national samples [49]. To address these objectives, we use multilevel logistic regression models that account for individuals nested within countries, using data from 32 nationally representative samples.

## Methods

### Data and Sample

The study combines individual-level and country-level data. Individual-level data were drawn from the Health and Health Care II module of the ISSP, collected between 2021 and 2024 across 32 countries [49-51]. The ISSP is a long-running cross-national collaboration that provides harmonized survey data based on probabilistic national samples of individuals aged 16 years and older. The integrated dataset used in this study includes countries from Europe, the Americas, Asia, Africa, and Oceania, allowing for comparative analysis across a diverse range of institutional and socioeconomic contexts. National sample sizes ranged from 1001 in Mexico to 3349 in Switzerland, yielding an initial sample of 48,302 respondents. Of these, 52.2% (25,213/48,302) were female, 24.9% (12,027/48,302) had tertiary education, and the mean age was 47.9 (SD 17.6) years. Further details on country coverage and fieldwork timing are provided in Table S1 of Multimedia Appendix 1.

Missing data were present across several variables, resulting in the exclusion of approximately 26.3% (12,703/48,302) of the initial sample in the final analytical dataset (n=35,592). The highest rates of missingness were observed for attitudinal measures of perceived usefulness of the internet (3767/48,302, 7.8%) and reliability of online health information (4781/48,302, 9.9%). These missing values were largely driven by the ISSP survey design, in which respondents without internet access or those who selected “can’t choose” were coded as missing.

Because imputing values for respondents without internet access would be conceptually inappropriate, these cases were not imputed. For the remaining variables, item nonresponse was generally low. However, comparison of respondents included in the final analytical sample with those excluded due to missing data revealed systematic differences (Table S2 in Multimedia Appendix 1). Excluded respondents were more likely to be older, female, less educated, economically inactive, and in poorer health, consistent with established patterns of digital exclusion. This indicates that missingness was unlikely to be fully random.

Multiple imputation was considered but not implemented due to the structurally induced missingness in internet-related variables and the risk of introducing cross-national artifacts. Country-specific imputation was also not feasible due to small numbers of missing observations within countries, which led to model instability and convergence issues. Given the relatively low level of item nonresponse and the stability of results across sensitivity analyses (including models excluding respondents without internet access), listwise deletion was retained as a parsimonious analytical strategy.

### Measurements

#### OHISB

OHISB was measured using four items from the ISSP Health and Health Care II module. Respondents were asked how often during the past 12 months they had used the internet on any device (eg, computer, tablet, or smartphone) to search for (1) health or medical information for themselves or others; (2) information about a healthy lifestyle; (3) information related to anxiety, stress, or similar issues; and (4) vaccination information. Response categories ranged from “never or almost never” to “several times a week.”

Because the ISSP question captures general internet searching behavior rather than the use of specific platforms, it reflects a broad range of digital environments through which individuals may obtain health information, including diverse digital channels such as search engines, apps, social media, and online forums.

For the main analyses, a binary indicator was constructed to distinguish between respondents who reported any engagement in online health information seeking (“health onliners”) and those who did not (“health offliners”) [33]. Respondents who indicated “never or almost never, I do not have access to the internet” across all items were classified as health offliners, while those reporting any higher frequency on at least one item were classified as health onliners. This operationalization reflects the conceptualization of OHISB as a behavioral outcome, where the absence of online health information seeking indicates non-engagement regardless of the underlying reason.

At the same time, this definition combines individuals who are unable to access the internet (ie, constrained nonuse) with those who have access but do not seek health information online (ie, voluntary nonuse), introducing heterogeneity in the outcome. Sensitivity analyses excluding respondents without internet access yielded substantively similar results, indicating that the main findings are not driven solely by access constraints. Descriptive comparisons further showed that OHISB prevalence is consistently higher when restricted to individuals with internet access (Table S3 in Multimedia Appendix 1).

Additionally, the use of a binary indicator reflects considerations of cross-national measurement comparability. Prior research suggests that single-item measures of online health information seeking may lack measurement invariance across countries, whereas composite indicators provide more robust comparability in cross-national settings [52]. The combined binary measure used in this study therefore captures engagement across multiple domains while reducing potential bias arising from cross-national differences in item functioning.

#### Individual-Level Variables

Individual-level variables were selected to capture sociodemographic characteristics, health status, health care experiences, and psycho-motivational factors associated with OHISB.

Sociodemographic variables included gender (female=1), age groups (eight ten-year categories), education (eight-level degree classification based on International Standard Classification of Education [ISCED] categories), employment status, and partnership status. These variables reflect established dimensions of social stratification associated with digital inequality. Income and minority status were excluded due to cross-national data limitations. Ethnicity was inconsistently coded across countries and entirely missing for Switzerland, while income data were available only for the economically active population, which would have substantially reduced the analytical sample.

Health-related variables comprised indicators of health status and health behaviors, including the presence of health problems, smoking, alcohol consumption, physical activity, disability, subjective health, and well-being. These variables capture both objective and subjective aspects of health that may be associated with engagement in health information seeking.

Health care-related variables included frequency of doctor visits, satisfaction with health care use, unmet health care needs, and trust in health care providers and systems (eg, doctor trust, confidence in the health care system, satisfaction with health care, perceived quality, and access). These indicators reflect individuals’ experiences with and perceptions of health care systems, which may also be associated with their engagement in online health information seeking.

Finally, psycho-motivational factors included perceived usefulness of the internet for health-related purposes and perceived reliability of online health information. These variables capture individuals’ familiarity with digital environments and their evaluation of online information sources.

To account for temporal differences in data collection, fieldwork year (2021‐2024) was included as an individual-level control variable. Because countries participated in different waves of data collection, this variable also captures potential temporal variation associated with post-pandemic changes in digital health engagement (Table S1 in Multimedia Appendix 1 for country-level fieldwork timing). It was modeled as a grand-mean–centered continuous variable (year minus 2021) to capture linear temporal variation. Sensitivity analyses using alternative specifications (dummy-coded years and quadratic term) produced substantively similar results and did not improve model fit.

Full definitions, coding details, and descriptive statistics are provided in Tables S4a–S4c of Multimedia Appendix 1.

#### Country-Level Variables

Country-level variables were selected to capture structural characteristics of national contexts that may be associated with cross-national variation in OHISB. Building on prior research and the theoretical framework of this study, four domains were considered: health care system characteristics, digital infrastructure, socioeconomic development, and cultural context.

Indicators of health care system characteristics included measures of health care resources and system performance, such as the number of physicians [53-55] and hospitals per capita [56-63], travel time to health facility [64], life expectancy, healthy life expectancy [65,66], and selected health-related behaviors at the population level (eg, smoking [67-69] and alcohol consumption [70,71]). Evaluative indicators were derived from aggregated survey measures capturing trust in doctors and perceptions of the health care system, including trust, satisfaction, perceived quality, and accessibility.

Digital infrastructure was captured through indicators of internet access and internet use [72-74]. Socioeconomic development was measured using macroeconomic and human development indicators, including gross domestic product [75,76], the human development index [77,78], and health expenditure [79-81]. Cultural context was represented using Hofstede’s cultural dimensions (eg, individualism and power distance) [82].

Given the large number of correlated macro-level indicators and the relatively small number of countries, these variables were aggregated into composite indices using principal component analysis (PCA). This approach reduces dimensionality, limits multicollinearity, and captures broader structural dimensions rather than relying on highly collinear single indicators. The analysis yielded four components representing (1) health system evaluation and trust, (2) socioeconomic and health development, (3) cultural hierarchy–individualism, and (4) unhealthy lifestyle patterns.

The resulting components were standardized and included in the multilevel models as country-level variables. As a robustness check, we also estimated alternative models using selected individual country-level indicators in place of the composite indices. These specifications produced substantively similar results, supporting the robustness of the PCA-derived indices used in the main analysis. Correlations between country-level variables, factor loadings, and component diagnostics are reported in Tables S5–S6 in Multimedia Appendix 1. Detailed country-level data are provided in the Open Science Framework repository [83].

### Analytical Strategy

To examine associations between individual- and country-level factors and OHISB, we estimated multilevel logistic regression models with individuals nested within countries. This approach accounts for the hierarchical structure of the data and allows for the separation of within- and between-country variation.

We first estimated a null model (Model 0) with a random intercept for countries to assess between-country variation [84]. The intraclass correlation coefficient (ICC) was calculated using the latent variable approach, assuming a level-1 variance of π²/3. This provided an estimate of the proportion of total variance attributable to differences between countries.

Individual-level variables were then introduced stepwise: sociodemographic characteristics (Model 1), health-related factors (Model 2), health care-related variables (Model 3), and psycho-motivational factors (Model 4). Country-level variables were subsequently added to the full individual-level model to assess contextual effects. PCA-derived indices were first introduced in separate models and then combined in a final model if they improved model fit.

As a robustness check, country-level indicators were also examined individually in models including all individual-level covariates to assess consistency of effects and potential multicollinearity. These alternative specifications yielded substantively similar results (Table S9a–S9b in Multimedia Appendix 1).

Cross-level interactions between selected country-level indices and theoretically relevant individual-level variables were explored to assess whether individual-level associations varied across country contexts. Only statistically significant interactions were retained.

Model fit was evaluated using the Bayesian information criterion and likelihood-ratio tests [85]. The proportional change in variance (PCV) was used to assess the contribution of successive models.

Different centering strategies were applied to distinguish within- and between-country effects and to reduce cross-level confounding. For macro-level variables derived from aggregated individual data, individual-level variables were group-mean centered and country-level aggregates grand-mean centered. For externally sourced country-level variables, both levels were grand-mean centered [86].

For descriptive analyses, the original ISSP design weights were applied to ensure national representativeness. Multilevel models were estimated without rescaled weights; robustness checks using rescaled weights yielded substantively similar results, indicating that findings were not sensitive to weighting strategy [87,88].

Multicollinearity was assessed using variance inflation factors, with all values below conventional thresholds (maximum variance inflation factor=2.07; Table S7 in Multimedia Appendix 1). Model assumptions, including linearity of continuous variables in the logit, were assessed, with no substantial violations detected. All annotated code and replication materials are available in the Open Science Framework repository [83].

### Ethical Considerations

This study is based on a secondary analysis of data from the ISSP (ISSP 2021‐2024). The dataset is fully anonymized and contains no personally identifiable information. No new data collection involving human participants was conducted by the authors.

The original ISSP data collection was conducted in accordance with national and institutional ethical standards in each participating country. Participation was voluntary and informed consent was obtained from all respondents. In addition, ISSP survey instruments are reviewed and approved by the ISSP General Assembly with regard to scientific quality and ethical appropriateness, and all national datasets are anonymized prior to inclusion in the ISSP archive to prevent identification of individual participants [89].

The authors had permission to use the data for research purposes in accordance with ISSP data access policies.

As this study involved only secondary analysis of fully anonymized data and did not involve interaction with human participants or access to identifiable personal data, it did not constitute human participants research as defined by applicable research ethics standards. Therefore, formal ethics approval was not required. No compensation was provided to participants in the context of this secondary analysis.

All analyses were conducted in accordance with established principles of research integrity and ethical data handling, including the Code of Ethics for Researchers of the Czech Academy of Sciences [90] and internationally recognized guidelines for secondary data use. Data privacy and confidentiality were maintained throughout the study.

## Results

### Descriptive Findings

**Figure 1.**
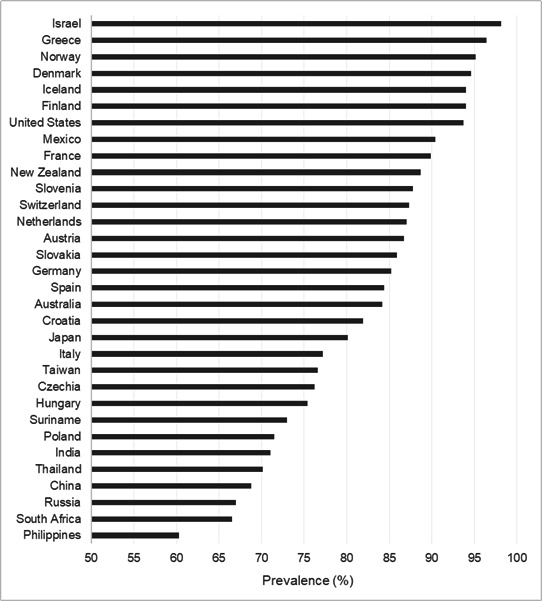
Prevalence of online health information–seeking behavior across countries (ranked), based on weighted data.

The prevalence of OHISB varied substantially across countries, ranging from 60.3% (1085/1787) in the Philippines to 98.1% (1164/1186) in Israel (Figure 1). Countries with lower levels of internet penetration generally exhibited lower prevalence, whereas countries with near-universal access showed consistently high levels of engagement.

When restricted to individuals with internet access, prevalence levels were higher across all countries, with particularly large differences observed in lower-access contexts (Table S3 in Multimedia Appendix 1).

### Individual-Level Associations

A null model indicated substantial between-country variation in OHISB. The ICC was 0.177 (95% CI 0.115‐0.264), corresponding to approximately 18% of the total variance attributable to differences between countries.

Sequential inclusion of individual-level variables improved model fit. Individual-level factors explained approximately one quarter of the between-country variance in OHISB (Table S8 in Multimedia Appendix 1).

No significant overall temporal effects were observed; fieldwork year was not systematically associated with OHISB.

**Figure 2.**
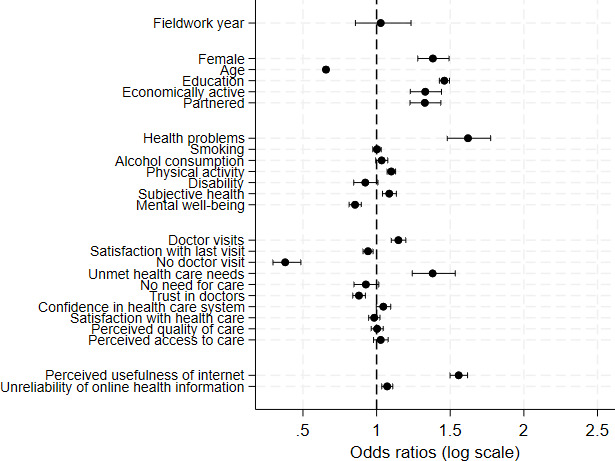
Odds ratios with 95% CIs for individual-level factors associated with online health information–seeking behavior. Estimates are derived from multilevel logistic regression models including all individual-level variables. Odds ratios greater than 1 indicate a higher likelihood of engaging in online health information–seeking behavior.

Sociodemographic factors showed consistent patterns. Individuals with higher education had significantly higher odds of OHISB (odds ratio [OR] 1.46, 95% CI 1.43‐1.49), while older respondents showed lower odds compared to younger age groups (OR 0.66, 95% CI 0.64‐0.67). Women reported higher engagement than men (OR 1.38, 95% CI 1.28‐1.49). Being partnered or economically active also had modest positive effects, as shown in Figure 2.

Health-related characteristics were also associated with OHISB. Individuals reporting poorer subjective health (OR 1.09, 95% CI 1.04‐1.13), the presence of health problems (OR 1.62, 95% CI 1.48‐1.77), and lower levels of well-being (OR 0.85, 95% CI 0.81‐0.90) were more likely to seek health information online. Health behaviors such as smoking, alcohol consumption, and physical activity showed more modest and less consistent associations.

Health care-related variables revealed that individuals with more frequent contact with health care services (OR 1.15, 95% CI 1.10‐1.20), unmet health care needs (OR 1.38, 95% CI 1.24‐1.53), and higher levels of trust in the health care system (OR 1.05, 95% CI 1‐1.10) were more likely to engage in OHISB. In contrast, individuals reporting higher satisfaction with their most recent doctor visit or with health care overall were less likely to seek health information online.

Psycho-motivational factors exhibited some of the strongest associations. Perceiving the internet as a useful health-related tool (OR 1.56, 95% CI 1.50‐1.62) and higher perceived reliability of online health information (OR 1.07, 95% CI 1.04‐1.11) were both strongly associated with a greater likelihood of OHISB.

### Country-Level Factors

Country-level structural conditions were associated with variation in OHISB across countries (Table 1). In particular, higher levels of socioeconomic and health development were linked to a greater likelihood of OHISB, with a one standard deviation increase in the index corresponding to substantially higher odds (OR=1.99, 95% CI 1.63‐2.45), with a PCV of 63%.

**Table 1.** Associations between country-level factors and online health information–seeking behavior.

Model	Country-level factor	OR^a^^,^^b^ (95% CI)	*P* value	BIC^c^	PCV^d^
Individual model	—^e^	—	—	20737.6	—
Health system evaluation	—	1.24 (0.94-1.63)	.13	20745.9	7
Socioeconomic and health development	—	1.99 (1.63-2.45)	<.001	20719.3	63
Cultural hierarchy–individualism index	—	1.89 (1.51-2.37)	<.001	20721.2	59
Unhealthy lifestyle	—	1.13 (0.83-1.54)	.42	20747.3	3
Final model	Socioeconomic and health development	1.52 (1.15-2)	.003	20726.0	76
	Cultural hierarchy–individualism index	1.37 (0.99-1.89)	.06	—	—

a OR: odds ratio.

b Note: Odds ratios represent the change in odds associated with a one SD increase in each country-level index. Proportional change in variance indicates the proportion of between-country variance explained relative to the individual-level model.

c BIC: Bayesian information criterion.

d PCV: proportional change in variance.

e Not available.

The cultural hierarchy–individualism index was also positively associated with OHISB (OR 1.89, 95% CI 1.51‐2.37).

In contrast, health system evaluation and unhealthy lifestyle patterns showed weak or non-significant associations with OHISB and did not substantially improve model fit. However, the health system evaluation index accounted for a small proportion of between-country variance (PCV=7%) (Table 1 and Tables S10a–S10b in Multimedia Appendix 1).

When both indices were included simultaneously, socioeconomic and health development remained associated with OHISB (OR 1.52, 95% CI 1.15‐2.00), whereas the association of cultural orientation was attenuated and no longer statistically significant (OR 1.37, 95% CI 0.99‐1.89). Including the cultural index provided only limited additional improvement in model fit beyond socioeconomic and health development, increasing the explained between-country variance from 63% to 76% (Table 1 and Table S11 in Multimedia Appendix 1).

### Cross-Level Interactions

Exploratory analyses identified cross-level interactions between selected country-level indices and individual-level variables (Table S12 in Multimedia Appendix 1).

**Figure 3.**
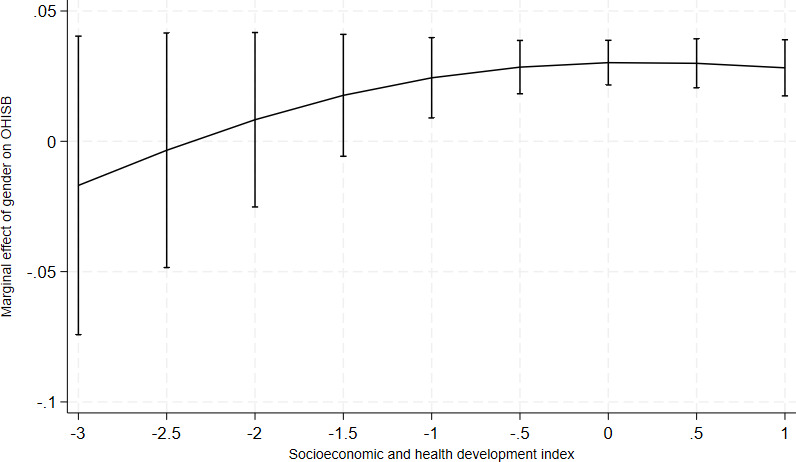
Cross-level interaction between socioeconomic and health development and gender: marginal effects on OHISB with 95% CIs. Predicted probabilities are derived from multilevel logistic regression models including individual-level variables and the socioeconomic and health development index. Error bars indicate 95% CIs. OHISB: online health information–seeking behavior.

First, the effect of gender differed by national development. The gender gap in OHISB, favoring women, increased with higher levels of socioeconomic and health development (OR 1.17, 95% CI 1.03‐1.31). As illustrated in Figure 3, gender differences were minimal in low-development contexts but became substantially larger in highly developed countries, where women were markedly more likely than men to seek health information online.

**Figure 4.**
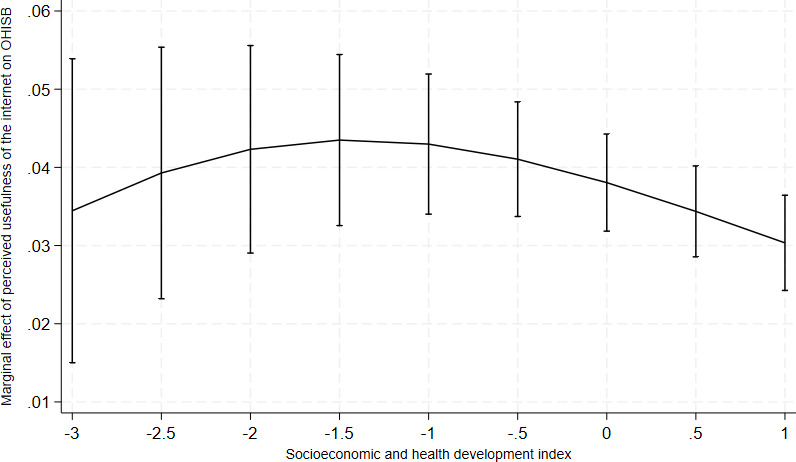
Cross-level interaction between socioeconomic and health development and perceived usefulness of the internet: marginal effects on online health information–seeking behavior with 95% CIs. Predicted probabilities are derived from multilevel logistic regression models including individual-level variables and the socioeconomic and health development index. Error bars indicate 95% CIs. OHISB: online health information–seeking behavior.

Second, perceived usefulness of the internet showed a context-dependent association. Its relationship with OHISB generally strengthened with increasing levels of national development but was not strictly monotonic (OR 1.09, 95% CI 1.05‐1.14). As shown in Figure 4, the effect of perceived usefulness was positive across all contexts, peaked in moderately developed countries, and slightly attenuated at the highest levels of development.

### Robustness Checks

To evaluate the stability of the findings, a series of robustness checks were conducted.

First, all analyses were replicated after excluding respondents without internet access. This restriction also reduced missingness in psycho-motivational variables to below 5% (typically around 2%). The re-estimated models produced substantively identical results. The direction and significance of individual-level associations remained unchanged, and country-level effects were stable. Although between-country variance and the ICC increased slightly, the overall pattern of results was unaffected.

Second, all models were estimated with and without the combined ISSP weight (WEIGHT_COM). For weighted analyses, the weights were normalized within countries (mean=1) and applied at the individual level, while countries remained equally weighted. The results were substantively identical across specifications: coefficients remained stable in both magnitude and significance, and the pattern of between-country variation was unchanged.

Third, individual country-level indicators were examined separately in models including all individual-level variables to assess the robustness of contextual effects. These analyses yielded consistent results: key indicators related to socioeconomic development, digital access, and population health were positively associated with OHISB and explained a substantial share of the between-country variation.

## Discussion

### Principal Findings

This study shows that OHISB follows well-established social gradients in digital and health-related behavior. Across 32 countries, younger, more educated, and female respondents were more likely to search for health information online, consistent with prior research on generational, educational, and gendered patterns in digital engagement [4,18]. These findings align with broader research on digital inequality.

Health-related factors were also associated with OHISB. Individuals reporting health problems or poorer mental well-being were more likely to search for health information online, suggesting that OHISB may reflect both coping-related and self-management–oriented responses to health needs. This pattern is consistent with previous research showing that health concerns and perceived vulnerability are important motivations for online health information seeking [3,21].

While individuals with more frequent contact with health care services were more likely to engage in OHISB, higher satisfaction with health care experiences was associated with a lower likelihood of online health information seeking. This pattern suggests that online health information seeking may serve both complementary and substitutive functions. Individuals dissatisfied with health care encounters may be more likely to seek additional information online, either to verify medical advice or to compensate for unmet informational needs, whereas those with more satisfactory health care experiences may rely more on formal sources of care and information [91,92].

Psycho-motivational variables showed some of the strongest associations. Perceived usefulness of the internet and higher perceived reliability of online health information were both positively associated with OHISB, underscoring the importance of digital familiarity and trust in shaping engagement with online health resources. These findings are consistent with information-seeking models that emphasize perceived utility and credibility of information sources as relevant factors of health information behavior [22,24].

At the country level, socioeconomic and health development emerged as the most robust contextual factor associated with OHISB. Individuals in more developed contexts were more likely to engage in online health information seeking, suggesting that structural conditions such as digital infrastructure, educational attainment, and population health are associated with environments that facilitate engagement with online health resources. In addition, this factor accounted for a substantial share of between-country variation.

These results align with research on the digital divide, which emphasizes that inequalities in digital engagement are not solely determined by individual characteristics but are also embedded in societal contexts [12]. Even when individuals have access to the internet, differences in national development shape the opportunities and conditions under which online health information can be accessed and used.

Cultural context also showed a meaningful association with OHISB when considered separately, with higher levels of individualism associated with greater engagement in online health information seeking. This finding suggests that cultural norms related to autonomy, self-reliance, and individual responsibility for health may play a role in shaping health information behavior. However, when considered jointly with socioeconomic development, the association of cultural orientation was attenuated, likely reflecting the empirical overlap between cultural and structural dimensions, as more economically developed countries also tend to exhibit higher levels of individualism [37].

Health system evaluation and unhealthy lifestyle patterns were not statistically significantly associated with OHISB. However, the health system evaluation index accounted for a small proportion of between-country variance, which may reflect limited statistical power at the country level rather than the absence of contextual relevance.

The analysis of cross-level interactions suggests that structural conditions may be associated with variation in the strength of selected individual-level factors. In particular, gender differences were more pronounced in highly developed contexts, with women showing higher levels of engagement relative to men. Similarly, the association between perceived usefulness of the internet and OHISB was generally stronger in higher-development settings. These findings suggest that structural conditions may be associated with differences in behavioral patterns between population groups and in the salience of motivational factors. However, these findings should be interpreted with caution, given the relatively small number of country-level units.

By combining nationally representative data from 32 countries with a multilevel framework, this study extends prior descriptive cross-national research and provides a more integrated assessment of how individual- and country-level factors are jointly associated with OHISB.

### Limitations and Future Research

However, several limitations should be acknowledged. First, the measurement of OHISB is necessarily broad and does not capture the heterogeneity of online health information seeking. The binary classification of respondents into health-onliners and health-offliners aggregates a wide range of behaviors, including searching for information related to prevention, symptoms, treatment, and mental health. These different forms of engagement may reflect distinct motivations and stages of health-related decision-making, ranging from primary prevention to disease management [93]. This aggregation may also obscure differences in the frequency and intensity of online health information seeking, which may vary systematically across social groups and country contexts. Although more fine-grained measures could capture additional behavioral variation, their cross-national comparability is limited [52]. Future research may benefit from examining specific domains of OHISB in more homogeneous contexts where measurement comparability can be more readily established.

Second, the binary operationalization of OHISB does not distinguish between structurally constrained nonseekers (eg, individuals without internet access) and motivationally disengaged non-seekers who have access but choose not to seek health information online. Although sensitivity analyses excluding respondents without internet access yielded substantively similar results, suggesting that the main findings are not driven solely by access barriers, these groups may differ in underlying mechanisms and responsiveness to policy interventions.

Third, the data do not allow differentiation between specific platforms or sources of online health information. Individuals may rely on diverse digital environments, including search engines, apps, social media, and online forums, which differ in credibility, accessibility, and informational quality. This heterogeneity may influence both the likelihood of seeking information and the potential benefits derived from it, but cannot be directly assessed in this study. Future research could also examine whether the observed patterns extend to emerging forms of digital health engagement, such as telemedicine, digital therapeutics, or AI-assisted health tools.

Fourth, the cross-sectional design of the study precludes causal interpretations of the observed associations. Relationships between OHISB and individual-level factors, particularly psycho-motivational variables such as perceived usefulness, may be bidirectional [94,95], and reverse causality cannot be ruled out.

Fifth, although the analysis includes a large number of countries, the number of level-2 units remains relatively limited for detecting complex cross-level interactions [84]. In addition, cross-level interactions were explored across multiple specifications, which increases the risk of Type I error. As a result, findings related to contextual moderation should be considered tentative.

Sixth, the analysis relies on listwise deletion, which resulted in the exclusion of a substantial proportion of cases. Although additional analyses indicated that the main findings were robust across alternative specifications, the assumption that missing data are random cannot be fully verified, and some degree of selection bias may remain. In particular, excluded respondents were more likely to belong to socially disadvantaged groups, which may lead to a conservative estimate of inequalities in OHISB.

Finally, the data were collected between 2021 and 2024, a period marked by the COVID-19 pandemic and its aftermath. Changes in health care access and increased reliance on digital resources during this period may have influenced patterns of online health information seeking. Because countries participated in different data collection waves, country-specific temporal variation related to differences in fieldwork timing cannot be fully ruled out. In addition, the rapid emergence and growing use of AI-assisted health information tools postdates much of the data collection and may affect the generalizability of the findings to current digital health environments.

Future research should extend these findings using longitudinal or repeated cross-sectional designs to better capture temporal dynamics in digital health engagement and strengthen causal inference. More granular macro-level indicators, such as digital skills distributions, health literacy environments, institutional trust, and digital governance, may help to identify additional contextual mechanisms underlying cross-national differences. Future research should also consider regulatory and legislative contexts shaping digital health information environments, including policies governing online health content and the control of misinformation.

### Practical Implications

The findings have several practical implications for efforts to reduce digital health inequalities. First, they highlight that improving access to digital health information alone is insufficient. Structural conditions, such as socioeconomic development, digital infrastructure, and population health, are associated with levels of engagement with online health resources. Policies aimed at reducing digital health inequalities should therefore combine improvements in access with broader investments in education, digital skills, and health literacy.

Second, the findings suggest that contextual conditions related to institutional trust, health system performance, and broader structural development may be relevant for engagement with online health information. This indicates that strengthening trust in health institutions and ensuring the availability of reliable, high-quality online health information may support more effective engagement with digital health resources.

Third, the findings on cross-level interactions indicate that the strength of individual-level associations may vary across contexts. In particular, gender differences and the role of perceived usefulness were more pronounced in more developed settings. These patterns suggest that differences in engagement between population groups may vary across contexts, indicating that interventions should be sensitive to contextual conditions rather than assuming uniform patterns across countries.

Finally, given the increasing importance of digital health technologies, including telemedicine and AI-driven tools, efforts to promote equitable digital health engagement should address both individual-level barriers and structural inequalities. As these technologies continue to evolve rapidly, they may further reshape how individuals access and evaluate health information, highlighting the need for policies that support both digital inclusion and the quality and trustworthiness of online health content. A comprehensive approach that integrates digital inclusion, health system development, and trust-building may be necessary to ensure that the benefits of digital health are distributed more evenly across populations.

### Conclusions

This study demonstrates that OHISB is associated with both individual characteristics and broader structural conditions. By integrating a large and geographically diverse set of countries within a multilevel framework, this study provides a more comprehensive assessment of how individual- and country-level factors jointly contribute to cross-national variation in digital health engagement. These findings highlight that digital health inequalities are embedded in structural contexts and underscore the importance of addressing both individual capabilities and broader contextual conditions to support more equitable engagement with digital health information.

## Supplementary material

10.2196/88110 Multimedia Appendix 1 Supplementary tables presenting detailed sample characteristics, descriptive statistics, country-level indicators, and full results of multilevel regression and sensitivity analyses.

## Abbreviations

AI: artificial intelligence
ICC: intraclass correlation coefficient
OHISB: online health information–seeking behavior
OR: odds ratio
PCA: principal component analysis
PCV: proportional change in variance
