# Supplementary material for: Structural Inequalities in Online Health Information Seeking: Cross-National Multilevel Study
Source: J Med Internet Res. 2026 May 19;28:e88110. doi: 10.2196/88110 (PMC13190103; doi:10.2196/88110)
Supplement: Multimedia Appendix 1 [file jmir-v28-e88110-s001.pdf]

Structural Inequalities in Online Health Information Seeking: A Cross-National Multilevel Study

Supplementary Materials

Abstract

Background: Online health information–seeking behavior (OHISB) has become an increasingly common component of contemporary health self-management. Individuals use a wide range of digital sources, including websites, social media platforms, and mobile applications, to obtain health-related information. However, substantial disparities persist in who seeks health information online and which populations benefit from digital health resources. While previous research has largely focused on individual-level determinants, cross-national evidence on structural influences remains limited.

Objective: This study aims to (1) assess between-country variation in OHISB, (2) examine associations between individual-level characteristics and OHISB, and (3) investigate how country-level structural conditions are associated with cross-national differences in OHISB, net of individual-level characteristics.

Methods: Data were drawn from the Health and Health Care II module of the International Social Survey Programme (ISSP 2021–2024; N = 35,592; 32 countries). OHISB was measured as any use of the internet to search for health-related information during the past 12 months. Multilevel logistic regression models were estimated. Country-level indicators were reduced using principal component analysis into four composite indices. Robustness checks included analyses excluding respondents without internet access and models incorporating survey weights.

Results: OHISB varied substantially across countries (intraclass correlation coefficient = 0.177). At the individual level, younger age, higher education, female respondents, recent health problems, doctor visits, unmet medical needs, and perceived usefulness of the internet were associated with higher odds of OHISB. At the macro level, the socioeconomic and health development showed the strongest association (OR = 1.52 per SD, P = .003) and explained a substantial share of between-country variation. Cultural hierarchy–individualism was associated with OHISB in separate models but attenuated when adjusted for development. Cross-level interactions indicated that the gender gap and the role of perceived usefulness were more pronounced in higher-development contexts, although these findings were exploratory.

Conclusions: OHISB is associated with both individual characteristics and broader structural conditions. Socioeconomic and health development appears to play a key contextual role in shaping cross-national differences in digital health engagement, highlighting the importance of addressing both individual and structural dimensions of digital health inequalities.

Keywords: online health information seeking; digital health; digital divide; multilevel analysis; cross-national comparison; socioeconomic development; structural determinants; health inequalities; ISSP

DATA & SAMPLE

Table S1. Country sample characteristics

Countries	Country - code	Number of respondents	Fieldwork year
Australia	AU	1,050	2021 - 2022
Austria	AT	1,546	2024
China	CN	2,689	2021
Taiwan	TW	1,604	2021 - 2022
Croatia	HR	1,101	2021
Czech Republic	CZ	1,262	2022
Denmark	DK	1,672	2021
Finland	FI	1,002	2022
France	FR	1,584	2022
Germany	DE	1,744	2021
Greece	GR	1,694	2022
Hungary	HU	1,008	2021
Iceland	IS	1,086	2022 - 2023
India	IN	1,683	2023
Israel	IL	1,187	2023
Italy	IT	1,138	2022 - 2023
Japan	JP	1,453	2021
Mexico	MX	1,001	2024
Netherlands	NL	1,269	2021
New Zealand	NZ	1,135	2022
Norway	NO	1,518	2021 - 2022
Philippines	PH	1,800	2021
Poland	PL	1,098	2022
Russia	RU	1,597	2022
Slovakia	SK	1,013	2021 - 2022
Slovenia	SI	1,020	2021
South Africa	ZA	2,829	2021
Spain	ES	2,059	2024
Suriname	SR	1,468	2022 - 2024
Switzerland	CH	3,349	2021
Thailand	TH	1,497	2021
United States	US	1,146	2022 - 2023
Total		48,302	

Source: ISSP Health and Health Care II (2021–2024). Authors' calculations.

Table S2. Comparison of included and excluded respondents

Variable	Excluded from analytical sample (n=12,609)	Included in analytical sample (n=35,592)	P value
Sex, n (%)			
Male	45.64	46.90	.015
Female	54.36	53.10	.015
Age (SD)	51.66 (20.97)	48.11 (16.64)	<.001
Education, n (%)			
No formal education	6.65	2.14	<.001
Primary	12.7	4.96	<.001
Lower secondary	19.22	13.74	<.001
Upper secondary	31.22	33.56	<.001
Post secondary	2.83	3.50	<.001
Short-cycle tertiary	7.84	10.41	<.001
Lower level tertiary	11.57	17.40	<.001
Upper level tertiary	7.07	12.82	<.001
Post tertiary	0.89	1.47	<.001
Partnered, n (%)	64.64	69.12	<.001
Economically active, n (%)	49.38	62.19	<.001
Subjective health, mean (SD)	2.96 (1.02)	3.17 (0.97)	<.001
Well-being score, mean (SD)	3.96 (1.01)	4.04 (0.94)	<.001

Source: *ISSP Health and Health Care II (2021–2024)*. Authors' calculations.

Note. Excluded respondents were defined as cases not retained in the final analytical sample due to missing values in one or more variables. Percentages are column percentages. P values are based on chi-square tests (categorical variables) and two-sample t tests (continuous variables).

DESCRIPTIVES

Table S3. Prevalence of online health information–seeking behavior (OHISB) by country

**Countries**	**Health-onliners**	**Health-offliners**	**People who do not have internet access**	**Missing values**	OHIS in total population	OHIS in the population with internet access
Philippines	60.3	5.6	33.4	0.7	60.3	91.5
South Africa	66.5	15.9	17.3	0.4	66.5	80.7
Russia	67	24.5	8.5	0	67	73.3
China	68.8	9.7	21.5	0.1	68.8	87.7
Thailand	70.1	4	25.9	0.1	70.1	94.6
India	71	18.3	9.9	0.7	71	79.5
Poland	71.5	0.4	28	0.1	71.5	99.4
Suriname	73	13.9	9.6	3.5	73	84
Hungary	75.4	17.3	7.2	0.2	75.4	81.3
Czech Republic	76.2	14	9.8	0	76.2	84.5
Taiwan	76.6	10.5	12.8	0.1	76.6	88
Italy	77.2	15.9	6.8	0.1	77.2	82.9
Japan	80.1	8.5	9.6	1.9	80.1	90.4
Croatia	81.9	14.1	4	0	81.9	85.3
Australia	84.2	9.6	2.9	3.3	84.2	89.7
Spain	84.4	8.5	5.9	1.3	84.4	90.8
Germany	85.2	7.3	7	0.5	85.2	92.1
Slovakia	85.9	8.7	4	1.4	85.9	90.8
Austria	86.7	9	4.2	0	86.7	90.6
Netherlands	87	12.5	0.2	0.3	87	87.4
Switzerland	87.3	10.2	2.4	0.1	87.3	89.6
Slovenia	87.8	6.9	4.8	0.4	87.8	92.7
New Zealand	88.7	8.7	2.5	0.1	88.7	91
France	89.9	9.7	0	0.4	89.9	90.3
Mexico	90.4	5.3	4.1	0.2	90.4	94.5
United States	93.7	5.4	0.4	0.4	93.7	94.5
Finland	94	4.6	1.4	0	94	95.3
Iceland	94	5.5	0.3	0.2	94	94.4
Denmark	94.6	4.2	0.4	0.7	94.6	95.7
Norway	95.1	4	0	0.9	95.1	96
Greece	96.4	3.6	0	0	96.4	96.4
Israel	98.1	1.5	0.3	0.1	98.1	98.5

Source: ISSP Health and Health Care II (2021–2024). Weighted using original ISSP design weights. Authors' calculations.

Table S4a. Descriptive statistics of the individual-level variables

**Variables**	**Range and categories**	**Mean**	**SD**	**%**	**Missing values in %**	**Association with health-onliners** rho/tau-b
Health-onliners	0 - 1 (composite score)			81.4	0.5	
Sociodemographic variables						
**Gender**	0 (men) - 1 (women) #rec			52.2	0.3	0.028^{***}
**Age**<br>**Age cohorts**	16 - 111 (in years)<br>1 (up to 20 years), 2 (21 – 30), 3 (31 to 40), 4 (41 to 50), 5 (51 to 60), 6 (61 to 70), 7 (71 to 80), 8 (over 80 years)	47.95<br>4.24	17.60<br>1.78		0.7	-0.274^{***}<br>-0.268^{***}
**Education**	0 (no formal education), 1 (primary education), 2 (lower education), 3 (upper education), 4 (post secondary, non-tertiary), 5 (short-cycle tertiary), 6 (lower level tertiary, BA), 7 (upper level tertiary, MA), 8 (Ph.D., Post Tertiary Specialization)	3.70	1.96		1.6	0.294^{***}
**Employment status**	0 (never had paid work or currently not in paid work, paid work in the past), 1 (currently in paid work) #rec			57.7	2.2	0.199^{***}
**Partnership**	0 (no partner – divorced, widowed or never married & not cohabitating), 1 (have a partner) #rec			64.9	2.4	0.055^{***}
Health-related factors						
**Health problems** (Did you experienced any health issues, bodily aches, or pain in the past four weeks?)	0 (past 4 weeks never felt health problems, bodily aches or pains), 1 (past 4 weeks from seldom to very often felt health problems, bodily aches or pains), composite score			78.9	0.7	0.092^{***}
**Smoking** (Do you smoke cigarettes, and if so, how many per day?)	1 (do not smoke cigarettes and never did), 2 (do not smoke now but smoked in the past), 3 (smoke 1-5 cigarettes per day), 4 (smoke 6-10 cigarettes per day), 5 (smoke 11-20 cigarettes per day), 6 (smoke 21-40 cigarettes per day), 7 (smoke more than 40 cigarettes per day)	1.88	1.33		1.7	-0.011^{***}

Table S4b. Descriptive statistics of the individual-level variables (continued)

Variables	Range and categories	Mean	SD	%	Missing values in %	Association with health-onliners rho/tau-b
**Alcohol use** (How often do you drink four or more alcoholic drinks on the same day?)	1 (never), 2 (once a month or less often), 3 (several times a month), 4 (several times a week), 5 (daily)	1.71	0.97		1.9	0.036***
**Physical activity** (How often do you engage in physical activity for at least 20 minutes that makes you sweat or breathe heavily?)	1 (never), 2 (once a month or less often), 3 (several times a month), 4 (several times a week), 5 (daily)	3.02	1.36		2.6	0.138***
**Disability** (Long-standing illness/ chronic condition/ disability)	0 (no) -1 (yes) #rec			29.6	1.2	-0.067***
**Subjective health**	1 (poor), 2 (fair), 3 (good), 4 (very good), 5 (excellent) #rec	3.10	0.99		1.1	0.092***
**Mental wellbeing** (Past 4 weeks: felt unhappy and depressed, lost confidence, not overcome problems) / items adapted from validated instruments such as SF-36, GHQ-12	1 - 5 (average index) Higher scores indicate better wellbeing.	3.99	0.98		0.7	-0.090***
Healthcare-related factors						
**Doctor visits** (During the past 12 months, how often did you visit or were visited by a doctor?)	1 (never) - 5 (very often)	2.37	1.04		1.2	0.034***
**Satisfaction with doctor visits** (How satisfied or dissatisfied were you with the treatment you received during your last visit?)	1 (completely dissatisfied) - 7 (completely satisfied) #rec	5.04	1.61		2.2	0.043***
**No doctor visit**	0 (doctor visit) 1 (no doctor visit)			4.4	2.4	-0.082***
**Unmet healthcare needs** (During the past 12 months did it ever happen that you did not get the medical treatment you needed because…)	0 (those who did not need medical treatment or those who received medical treatment in the last 12 months, if they needed it), 1 (those, who did not get the medical treatment, if they needed last 12 months for whatever reason) (composite score)			22.0	0.5	0.070***

Table S4c. Descriptive statistics of the individual-level variables (continued)

Variables	Range and categories	Mean	SD	%	Missing values in %	Association with health-onliners<br>rho/tau-b
**Not need medical treatment**	0 (any unmet needs), 1 (did not need medical treatment)			28.3	0.5	-0.031***
**Trust in doctors**<br>(All things considered, doctors can be trusted.)	1 (strongly agree) – 5 (strongly disagree) #rec	3.77	0.86		1.5	-0.030***
**Confidence in healthcare system**<br>(In general, how much confidence do you have in the healthcare system in [country]?)	1 (no confidence at all) - 5 (complete confidence) #rec	3.34	0.96		1.4	0.002
**Satisfaction with healthcare system**<br>(In general, how satisfied or dissatisfied are you with the healthcare system in [country]?)	1 (completely dissatisfied) - 7 (completely satisfied) #rec	4.74	1.29		1.9	-0.027***
**Perceived quality of care**<br>(How likely is it that if you become seriously ill, you would receive the best treatment available in [country]?)	1 (it's certain I would not get), 2 (it's likely I would not get), 3 (equal chance of getting or not getting), 4 (it's likely I would get), 5 (it's certain I would get) #rec	3.59	1.05		4.1	0.014**
**Perceived access to care**<br>(In [country], do you think it is easier or harder for rich people than for poor people to access healthcare?)	1 (much easier/much harder), 2 (somewhat easier/somewhat harder), 3 (about the same) #rec	1.67	0.76		3.1	0.016***
Psycho-motivational factors						
**Usefulness of internet**<br>(The internet is useful for helping people decide if their symptoms are serious enough to see a doctor. The internet is useful for checking if the doctor is giving appropriate advice.)	1 - 5 (average index)	3.00	1.00		7.5	0.076***
**Unreliability of information**<br>(It is not easy to distinguish between reliable and unreliable health information on the internet.)	1 (strongly disagree) - 5 (strongly agree) #rec	3.71	1.02		9.6	0.065***

Source: ISSP Health and Health Care II (2021–2024). Weighted using original ISSP design weights.
Note. Variables marked with "#rec" were recoded from original scales; ****P*<.001.

COUNTRY-LEVEL VARIABLES

Table S5. Correlations between country-level variables

	Conf	Satis	Qual	Access	Phy	Hospi	Travel	Trust	Life	H-Life	Tob	Alcoh	Power	Indivi	Iaccess	Iusage	GDP	HDI	H-exp
Conf																			
Satis	.86																		
Qual	.41	.63																	
Access	.39	.52	.72																
Phy	.13	-.02	.23	.18															
Hospi	.17	.13	-.14	-.24	.16														
Travel	-.23	-.24	-.39	-.20	-.31	-.21													
Trust	.84	.79	.43	.46	.31	.18	-.37												
Life	.41	.35	.37	.27	.69	.31	-.34	.51											
H-Life	.42	.33	.30	.23	.68	.32	-.27	.52	.99										
Tob	-.18	-.38	-.32	-.16	.36	.01	-.22	-.05	.16	.15									
Alcoh	.11	.01	.21	.13	.50	.16	-.33	.06	.28	.21	.27								
Power	-.30	-.43	-.60	-.42	-.46	.01	.40	-.37	-.52	-.46	.19	-.29							
Indivi	.40	.31	.44	.40	.78	.23	-.22	.43	.73	.73	.12	.58	-.62						
Iaccess	.13	.06	.24	.16	.61	-.10	-.20	.20	.67	.64	.31	.54	-.46	.65					
Iusage	.17	.14	.41	.22	.64	-.04	-.24	.21	.66	.64	.12	.56	-.62	.74	.91				
GDP	.36	.32	.48	.31	.76	.20	-.36	.42	.83	.80	.11	.48	-.73	.87	.77	.87			
HDI	.32	.26	.45	.26	.80	.22	-.45	.43	.88	.86	.17	.51	-.66	.84	.81	.86	.96		
Hexpen	.21	.19	.39	.24	.56	.18	-.42	.23	.53	.46	.20	.61	-.62	.73	.61	.68	.81	.75	

Source: ISSP Health and Health Care II (2021–2024) and external data sources. Author's calculations.

Note. All correlations are statistically significant at $P < .001$.

Table S6. Principal component analysis (PCA) of country-level indicators

Macro-level index	Included variables	Eigenvalue (1st component)	Explained variance (%)	Factor loadings	VIF
**Health system evaluation and trust**		3.44	68.9		
	Confidence in the healthcare			0.47	
	Satisfaction with healtcare			0.50	
	Perceived quality			0.41	
	Accessibility			0.39	
	Trust in doctors			0.47	
**Socioeconomic & health development**		6.22	77.7		4.11
	GDP per capita			0.39	
	HDI			0.40	
	Physician density			0.33	
	Internet access			0.34	
	Internet use			0.36	
	Life expectancy			0.36	
	Healthy life expectancy			0.35	
	Health expenditure (% GDP)			0.31	
**Cultural individualism–hierarchy**		1.62	81.2		3.50
	Power distance			-0.71	
	Individualism			0.71	
**Unhealthy lifestyle**		1.27	63.3		1.34
	Smoking prevalence			0.71	
	Alcohol consumption			0.71	

Note. PCA was conducted to derive composite indices from country-level indicators. Factor loadings represent correlations between variables and extracted components. Only the first principal component for each index is reported. All components were standardized (mean = 0, SD = 1) prior to inclusion in the multilevel models.

DIAGNOSTICS

Table S7. Variance inflation factors (VIF) for individual-level variables

Coefficients	Collinearity Statistics	
	Tolerance	VIF
Gender (female)	.901	1.110
Age (groups)	.683	1.464
Education	.888	1.126
Employment status	.830	1.205
Partnership (partner)	.886	1.128
Health problems	.831	1.203
Smoking	.900	1.111
Alcohol consumption	.892	1.121
Physical activity	.945	1.058
Disability	.758	1.320
Subjective health	.681	1.468
Mental wellbeing	.749	1.336
Doctor visits	.724	1.380
Satisfaction with last doctor visit	.763	1.310
Trust in doctors	.703	1.422
Perceived usefulness of internet	.933	1.072
Unreliability of information	.965	1.036
Unmet healthcare needs	.845	1.183
Confidence in healthcare	.581	1.721
Satisfaction with healthcare	.484	2.065
Perceived quality of care	.680	1.471
Perceived access to care	.940	1.064

Note. VIF values were calculated from a linear model including all individual-level predictors. All values were below conventional thresholds, indicating no evidence of problematic multicollinearity.

MAIN MODELS

Table S8. Multilevel logistic regression models: Individual-level variables

	M0			M1			M2			M3			M4		
**Variable**	**logit**	**SE**	**OR**	**logit**	**SE**	**OR (95% CI)**	**logit**	**SE**	**OR (95% CI)**	**logit**	**SE**	**OR (95% CI)**	**logit**	**SE**	**OR (95% CI)**
Filedwork year				0.036	(0.09)	1.04 (0.87,1.24)	0.025	(0.09)	1.03 (0.86, 1.22)	0.01	(0.09)	1.01 (0.85, 1.20)	0.027	(0.09)	1.03 (0.86, 1.23)
Gender (*female*)				0.361***	(0.04)	1.43 (1.34,1.54)	0.337***	(0.04)	1.40 (1.30, 1.51)	0.315***	(0.04)	1.37 (1.27, 1.48)	0.324***	(0.04)	1.38 (1.28, 1.49)
Age (*groups*)				-0.408***	(0.01)	0.66 (0.65,0.68)	-0.420***	(0.01)	0.66 (0.64, 0.67)	-0.428***	(0.01)	0.65 (0.64, 0.67)	-0.421***	(0.01)	0.66 (0.64, 0.67)
Education				0.362***	(0.01)	1.44 (1.40,1.47)	0.371***	(0.01)	1.45 (1.42, 1.48)	0.373***	(0.01)	1.45 (1.42, 1.49)	0.379***	(0.01)	1.46 (1.43, 1.49)
Employment status				0.253***	(0.04)	1.29 (1.19,1.39)	0.289***	(0.04)	1.34 (1.24, 1.44)	0.276***	(0.04)	1.32 (1.22, 1.43)	0.286***	(0.04)	1.33 (1.23, 1.44)
Partnership (*partner*)				0.270***	(0.04)	1.31 (1.21,1.41)	0.301***	(0.04)	1.35 (1.25, 1.46)	0.286***	(0.04)	1.33 (1.23, 1.44)	0.283***	(0.04)	1.33 (1.23, 1.44)
Health problems							0.578***	(0.05)	1.78 (1.63, 1.95)	0.486***	(0.05)	1.63 (1.49, 1.78)	0.483***	(0.05)	1.62 (1.48, 1.77)
Smoking							-0.006	(0.01)	0.99 (0.97, 1.02)	-0.003	(0.01)	1.00 (0.97, 1.02)	0.002	(0.01)	1.00 (0.97, 1.03)
Alcohol consumption							0.040*	(0.02)	1.04 (1.00, 1.08)	0.040*	(0.02)	1.04 (1.00, 1.08)	0.034	(0.02)	1.03 (0.99, 1.08)
Physical activity							0.099***	(0.01)	1.10 (1.08, 1.13)	0.096***	(0.01)	1.10 (1.07, 1.13)	0.095***	(0.01)	1.10 (1.07, 1.13)
Disability							0.028	(0.04)	1.03 (0.94, 1.12)	-0.075	(0.04)	0.93 (0.85, 1.01)	-0.08	(0.05)	0.92 (0.84, 1.01)
Subjective health							0.046*	(0.02)	1.05 (1.01, 1.09)	0.088***	(0.02)	1.09 (1.05, 1.14)	0.083***	(0.02)	1.09 (1.04, 1.13)
Mental wellbeing							-0.236***	(0.02)	0.79 (0.75, 0.83)	-0.176***	(0.02)	0.84 (0.80, 0.88)	-0.158***	(0.02)	0.85 (0.81, 0.90)
Doctor visits										0.134***	(0.02)	1.14 (1.10, 1.19)	0.138***	(0.02)	1.15 (1.10, 1.20)
Satisfaction with last doctor visit										-0.052**	(0.02)	0.95 (0.92, 0.98)	-0.060**	(0.02)	0.94 (0.91, 0.98)
No doctor visit										-0.950***	(0.12)	0.39 (0.30, 0.49)	-0.969***	(0.13)	0.38 (0.30, 0.49)
Unmet healthcare needs										0.353***	(0.05)	1.42 (1.28, 1.58)	0.322***	(0.05)	1.38 (1.24, 1.53)
Not need medical treatment										-0.082	(0.05)	0.92 (0.84, 1.01)	-0.076	(0.05)	0.93 (0.85, 1.02)
Trust in doctors										-0.108***	(0.02)	0.90 (0.86, 0.94)	-0.128***	(0.02)	0.88 (0.84, 0.92)
Confidence in healthcare										0.043	(0.02)	1.04 (1.00, 1.09)	0.045	(0.02)	1.05 (1.00, 1.10)
Satisfaction with healthcare										-0.003	(0.02)	1.00 (0.96, 1.04)	-0.016	(0.02)	0.98 (0.95, 1.02)
Perceived quality of care										0.004	(0.02)	1.00 (0.96, 1.05)	0.003	(0.02)	1.00 (0.96, 1.05)
Perceived access to care										0.008	(0.02)	1.01 (0.96, 1.06)	0.027	(0.02)	1.03 (0.98, 1.08)
Perceived usefulness of internet													0.443***	(0.02)	1.56 (1.50, 1.62)
Unreliability of information													0.070***	(0.02)	1.07 (1.04, 1.11)
Constant	2.229***	(0.15)		2.330***	(0.19)		2.361***	(0.22)		2.371***	(0.25)		0.859**	(0.27)	
Country variability	0.711***	(0.18)	2.04	0.648***	(0.17)	1.91	0.542***	(0.14)	1.72	0.553***	(0.14)	1.74	0.683***	(0.18)	1.98
ICC			0.178			0.164			0.141			0.144			0.172
AIC			25241.81			21737.18			21289.17			21058.04			20508.93
BIC			25258.77			21805.02			21416.37			21270.04			20737.89
BIC diff vs prev.			—			-3453.75			-388.65			-146.34			-532.15
-2LL			25237.81			21721.18			21259.17			21008.04			20454.93
PCV vs M0			—			0.090			0.239			0.222			0.039

Source: *ISSP 2021 and external sources of data*. Authors' calculations. (N = 35,592; 32 countries). Note. Odds ratios (ORs) are reported. *** P < .001, ** P < .01, * P < .05.

ROBUSTNESS – SINGLE INDICATORS

Table S9a. Sensitivity analysis: Country-level indicators entered separately

Category	Country-level factor	Odds Ratio (per 1 SD increase) (95% CI)	Odds Ratio [95% CI]	Logit	SE	*P* (>|z|)	Fdr-p	BIC	AIC	ΔBIC (vs. M4_base)	Country var	ICC	PCV
Individual model		—	—	—	—	—	—	20737.6	20508.64	—	0.68	0.17	—
**Public health & healthcare system**	Confidence in healthcare	1.07 (0.79, 1.45)	1.19 (0.54, 2.61)	0.17	0.40	.668	0.705	20747.89	20510.45	10.3	0.67	0.17	1%
	Satisfaction with healthcare	1.08 (0.79, 1.47)	1.16 (0.63, 2.16)	0.15	0.31	.633	0.705	20747.82	20510.38	10.2	0.67	0.17	1%
	Perceived quality of care	1.44 (1.11, 1.87)	2.72 (1.32, 5.62)	1.00	0.37	.007	0.01	20741.65	20504.22	4.1	0.54	0.14	19%
	Perceived access to care	1.34 (1.08, 1.65)	4.34 (1.49, 12.60)	1.47	0.54	.007	0.01	20743.81	20506.37	6.2	0.58	0.15	14%
	Physicians	1.77 (1.40, 2.22)	1.47 (1.26, 1.71)	0.39	0.11	<.001	<.001	20731.55	20494.11	-6.1	0.39	0.11	42%
	Number of hospitals	0.96 (0.70, 1.30)	1.00 (0.97, 1.02)	-0.003	0.01	.784	0.784	20510.55	20747.99	10.4	0.66	0.17	0.3%
	Travel time to health.	0.83 (0.62, 1.12)	0.97 (0.91, 1.02)	-0.03	0.03	.233	0.316	20746.56	20509.12	9.0	0.64	0.16	5%
	Trust in doctors	1.17 (0.93, 1.48)	2.10 (0.69, 6.37)	0.74	0.56	.189	0.276	20746.89	20509.46	9.3	0.64	0.17	4%
	Life expectancy	1.89 (1.41, 2.52)	1.11 (1.06, 1.16)	0.10	0.02	<.001	<.001	20727.29	20489.85	-10.3	0.34	0.09	51%
	Healthy life expectancy	1.89 (1.39, 2.56)	1.13 (1.06, 1.19)	0.12	0.03	<.001	<.001	20727.99	20490.56	-9.6	0.35	0.10	49%
	Tobacco use	1.09 (0.83, 1.44)	1.01 (0.97, 1.05)	0.01	0.02	.523	0.663	20747.66	20510.22	10.1	0.67	0.17	2%
	Alcohol consumption	1.11 (0.78, 1.59)	1.04 (0.91, 1.20)	0.04	0.07	.558	0.663	20747.55	20510.12	10.0	0.66	0.17	2%
**Cultural dimensions**	Power Distance	0.60 (0.45, 0.80)	0.98 (0.97, 0.99)	-0.02	0.006	<.001	<.001	20732.57	20495.14	-5.0	0.41	0.11	39%
	Individualism	1.90 (1.54, 2.33)	1.03 (1.02, 1.04)	0.03	0.004	<.001	<.001	20722.69	20485.25	-14.9	0.28	0.08	59%

Table S9b. Sensitivity analysis: Country-level indicators entered separately (continued)

Category	Variable	Odds Ratio (per 1 SD increase) [95% CI]	Odds Ratio [95% CI]	Logit	SE	*P* (>|z|)	Fdr-p	BIC	AIC	ΔBIC (vs. M4_base)	Country var	ICC	PCV
**Digital infrastructure**	Internet access	1.68 (1.38, 2.03)	1.04 (1.02, 1.05)	0.04	0.01	<.001	<.001	20734.73	20497.29	-2.9	0.42	0.11	38%
	Internet usage	1.80 (1.50, 2.16)	1.05 (1.03, 1.06)	0.05	0.01	<.001	<.001	20728.76	20491.33	-8.8	0.35	0.10	48%
**Socioeconomic development**	GDP (log)	2.00 (1.65, 2.43)	2.00 (1.65, 2.42)	0.69	0.10	<.001	<.001	20717.49	20480.05	-20.1	0.24	0.07	65%
	HDI	1.94 (1.51, 2.49)	1.07 (1.05, 1.10)	0.07	0.01	<.001	<.001	20722.92	20485.49	-14.7	0.28	0.08	59%
	Health expenditure	1.58 (1.17, 2.13)	1.18 (1.06, 1.32)	0.17	0.06	.003	0.006	20736.69	20499.26	-0.9	0.46	0.12	32%

Source: *ISSP Health and Health Care II (2021–2024) and external data sources*. Authors' calculations. (N = 35,592; 32 countries).

Note. Each country-level indicator was tested separately in models including all individual-level variables. P values were adjusted using the Benjamini–Hochberg false discovery rate (FDR) procedure. Continuous indicators were standardized; ORs represent one standard deviation increase.

PCA MODELS

Table S10a. Multilevel models with PCA-derived country-level indices (separate models)

	M5-health_sys			M6-socio_develop		
**Variables**	**Logit**	**rob. SE**	**OR (95% CI)**	**Logit**	**rob. SE**	**OR (95% CI)**
Fieldwork year	0.48	0.07	1.05 (0.92, 1.20)	0.11	0.06	1.11 (0.99, 1.26)
Gender (*female*)	0.33***	0.06	1.38 (1.23, 1.56)	0.33***	0.06	1.38 (1.22, 1.55)
Age (groups)	-0.42***	0.06	0.66 (0.58, 0.74)	-0.42***	0.06	0.65 (0.58, 0.74)
Education (*degree levels*)	0.38***	0.04	1.46 (1.36, 1.57)	0.38***	0.04	1.46 (1.36, 1.56)
Employment status *(active)*	0.28*	0.11	1.33 (1.06, 1.67)	0.28*	0.11	1.33 (1.06, 1.67)
Partnership *(partner)*	0.28***	0.06	1.33 (1.18, 1.49)	0.28***	0.06	1.33 (1.19, 1.49)
Health problems	0.48***	0.09	1.62 (1.34, 1.95)	0.48***	0.09	1.62 (1.35, 1.95)
Smoking	0.002	0.02	1.00 (0.97, 1.04)	0.002	0.02	1.00 (0.97, 1.04)
Alcohol use	0.03	0.02	1.03 (0.99, 1.09)	0.03	0.02	1.03 (0.99, 1.08)
Physical activity	0.09**	0.04	1.10 (1.03, 1.18)	0.09**	0.04	1.10 (1.02, 1.18)
Disability	-0.08	0.09	0.92 (0.78, 1.10)	-0.08	0.09	0.92 (0.78, 1.09)
Subjective health	0.08**	0.03	1.09 (1.02, 1.15)	0.08**	0.03	1.09 (1.02, 1.16)
Mental wellbeing	-0.16***	0.05	0.85 (0.78, 0.94)	-0.16***	0.05	0.85 (0.78, 0.94)
Doctor visits	0.14***	0.04	1.15 (1.06, 1.24)	0.14***	0.04	1.15 (1.06, 1.24)
Satisfaction with doctor visits	-0.06	0.03	0.94 (0.89, 1.00)	-0.06	0.03	0.94 (0.89, 1.00)
No doctor visit	-0.97**	0.31	0.38 (0.21, 0.70)	-0.97**	0.31	0.38 (0.21, 0.69)
Unmet healthcare needs	0.32**	0.11	1.38 (1.12, 1.70)	0.32**	0.11	1.39 (1.12, 1.71)
Not need medical treatment	-0.08	0.06	0.93 (0.83, 1.04)	-0.08	0.06	0.92 (0.83, 1.04)
Trust in doctors	-0.13***	0.03	0.88 (0.82, 0.94)	-0.13***	0.03	0.88 (0.82, 0.94)
Confidence in healthcare	0.04	0.05	1.05 (0.96, 1.14)	0.04	0.05	1.05 (0.96, 1.14)
Satisfaction with healthcare	-0.02	0.04	0.98 (0.91, 1.07)	-0.02	0.04	0.98 (0.91, 1.07)
Perceived quality of care	0.002	0.04	1.00 (0.93, 1.08)	0.002	0.04	1.00 (0.93, 1.08)
Perceived access to care	0.03	0.06	1.03 (0.90, 1.17)	0.03	0.06	1.03 (0.90, 1.16)
Perceived usefulness of internet	0.44***	0.04	1.56 (1.44, 1.68)	0.44***	0.04	1.56 (1.45, 1.68)
Unreliability of information	0.07*	0.03	1.07 (1.01, 1.13)	0.07*	0.03	1.07 (1.01, 1.13)
Country-level variables						
Health system evaluation and trust	0.21	0.14	1.24 (0.94, 1.63)			
Socioeconomic & health development				0.69***	0.10	1.99 (1.63, 2.45)
Others						
Constant	1.83	0.19		1.71	0.17	
Country variability	0.627	0.18		0.250	0.06	
ICC			0.16			0.07
AIC			20508.46			20481.89
BIC			20745.9			20719.33
BIC diff			8.3			-18.3
-2LL			-10226.23			-10212.95
PCV			0.07			0.63

Source: *ISSP Health and Health Care II (2021–2024) and external data sources.* Authors' calculations. (N = 35,592; 32 countries). Note. Each index was entered separately into the model.

Table S10b. Multilevel models with PCA-derived country-level indices (separate models) (continued)

	M7-culture			M8-lifestyle		
**Variables**	**Logit**	**rob. SE**	**OR (95% CI)**	**Logit**	**rob. SE**	**OR (95% CI)**
Fieldwork year	0.07	0.07	1.08 (0.93, 1.24)	0.03	0.06	1.03 (0.91, 1.17)
Gender (*female*)	0.33***	0.06	1.38 (1.23, 1.56)	0.33***	0.06	1.38 (1.23, 1.56)
Age (groups)	-0.42***	0.06	0.66 (0.58, 0.74)	-0.42***	0.06	0.66 (0.58, 0.74)
Education (*degree levels*)	0.38***	0.04	1.46 (1.36, 1.56)	0.38***	0.04	1.46 (1.36, 1.57)
Employment status *(active)*	0.28*	0.11	1.33 (1.06, 1.67)	0.28*	0.11	1.33 (1.06, 1.67)
Partnership *(partner)*	0.28***	0.06	1.33 (1.18, 1.49)	0.28***	0.06	1.33 (1.18, 1.49)
Health problems	0.48***	0.09	1.62 (1.34, 1.95)	0.48***	0.09	1.62 (1.35, 1.95)
Smoking	0.002	0.02	1.00 (0.97, 1.04)	0.002	0.02	1.00 (0.97, 1.04)
Alcohol use	0.03	0.02	1.03 (0.98, 1.08)	0.03	0.02	1.03 (0.99, 1.09)
Physical activity	0.09**	0.04	1.10 (1.03, 1.18)	0.09**	0.04	1.10 (1.03, 1.18)
Disability	-0.08	0.09	0.92 (0.78, 1.10)	-0.08	0.09	0.92 (0.78, 1.10)
Subjective health	0.08**	0.03	1.09 (1.02, 1.15)	0.08**	0.03	1.09 (1.02, 1.15)
Mental wellbeing	-0.16***	0.05	0.85 (0.78, 0.94)	-0.16***	0.05	0.85 (0.78, 0.94)
Doctor visits	0.14***	0.04	1.15 (1.06, 1.24)	0.14***	0.04	1.15 (1.06, 1.24)
Satisfaction with doctor visits	-0.06*	0.03	0.94 (0.88, 1.00)	-0.06	0.03	0.94 (0.89, 1.00)
No doctor visit	-0.97**	0.31	0.38 (0.21, 0.69)	-0.97**	0.31	0.38 (0.21, 0.70)
Unmet healthcare needs	0.32**	0.11	1.38 (1.12, 1.71)	0.32**	0.11	1.38 (1.12, 1.70)
Not need medical treatment	-0.08	0.06	0.93 (0.83, 1.04)	-0.08	0.06	0.93 (0.83, 1.04)
Trust in doctors	-0.13***	0.03	0.88 (0.82, 0.94)	-0.13***	0.03	0.88 (0.82, 0.94)
Confidence in healthcare	0.04	0.05	1.05 (0.96, 1.14)	0.04	0.05	1.05 (0.96, 1.14)
Satisfaction with healthcare	-0.02	0.04	0.98 (0.91, 1.07)	-0.02	0.04	0.98 (0.91, 1.07)
Perceived quality of care	0.002	0.04	1.00 (0.93, 1.08)	0.002	0.04	1.00 (0.93, 1.08)
Perceived access to care	0.03	0.06	1.03 (0.90, 1.16)	0.03	0.06	1.03 (0.90, 1.16)
Perceived usefulness of internet	0.44***	0.04	1.56 (1.44, 1.68)	0.44***	0.04	1.56 (1.45, 1.68)
Unreliability of information	0.07*	0.03	1.07 (1.01, 1.13)	0.07*	0.03	1.07 (1.01, 1.13)
Country-level variables						
Cultural hierarchy–individualism	0.64***	0.11	1.89 (1.51, 2.37)			
Unhealthy lifestyle				0.13	0.16	1.13 (0.83, 1.54)
Others						
Constant	1.76	0.19		1.81	0.20	
Country variability	0.274	0.06		0.658	0.18	
ICC			0.08			0.17
AIC			20483.75			20509.88
BIC			20721.18			20474.32
BIC diff			-16.4			9.7
-2LL			-10213.87			-10226.94
PCV			0.59			0.03

Source: *ISSP Health and Health Care II (2021–2024) and external data sources*. Authors' calculations. (N = 35,592; 32 countries). Note. Each index was entered separately into the model. *** P < .001, ** P < .01, * P < .05

Table S11. Multilevel model with combined PCA-derived indices

	M10 - Final model		
**Variables**	**Logit**	**rob. SE**	**OR (95% CI)**
Fieldwork year	0.10	0.07	1.11 (0.97, 1.26)
Gender (*female*)	0.32***	0.06	1.38 (1.23, 1.55)
Age (*cohorts*)	-0.42***	0.06	0.65 (0.58, 0.74)
Education (*degree levels*)	0.38***	0.04	1.46 (1.36, 1.56)
Employment status *(active)*	0.28*	0.11	1.33 (1.06, 1.67)
Partnership *(partner)*	0.28***	0.06	1.33 (1.18, 1.49)
Health problems	0.48***	0.09	1.62 (1.35, 1.95)
Smoking	0.002	0.02	1.00 (0.97, 1.04)
Alcohol use	0.03	0.02	1.03 (0.99, 1.08)
Physical activity	0.09**	0.04	1.10 (1.02, 1.18)
Disability	-0.08	0.09	0.92 (0.77, 1.09)
Subjective health	0.08**	0.03	1.09 (1.02, 1.15)
Mental wellbeing	-0.16***	0.05	0.85 (0.78, 0.94)
Doctor visits	0.14***	0.04	1.15 (1.06, 1.24)
Satisfaction with doctor visits	-0.06	0.03	0.94 (0.88, 1.00)
No doctor visit	-0.97**	0.31	0.38 (0.21, 0.69)
Unmet healthcare needs	0.32**	0.11	1.39 (1.12, 1.71)
Not need medical treatment	-0.08	0.06	0.93 (0.83, 1.04)
Trust in doctors	-0.13***	0.03	0.88 (0.82, 0.94)
Confidence in healthcare	0.04	0.05	1.05 (0.96, 1.14)
Satisfaction with healthcare	-0.02	0.04	0.98 (0.91, 1.07)
Perceived quality of care	0.002	0.04	1.00 (0.93, 1.08)
Perceived access to care	0.03	0.06	1.03 (0.90, 1.16)
Perceived usefulness of internet	0.45***	0.04	1.56 (1.45, 1.68)
Unreliability of information	0.07*	0.03	1.07 (1.01, 1.13)
Country-level variables			
Socioeconomic & health development	0.41**	0.14	1.52 (1.15, 2.00)
Cultural hierarchy–individualism	0.31	0.17	1.37 (0.99, 1.89)
Others			
Constant	1.72	0.18	
Country variability	0.221	0.05	
ICC			0.06
AIC			20480.11
BIC			20726.03
BIC diff (to M4_base)			-28.5
BIC diff (to M6)			-1.8
-2LL			-10211.06
PCV			0.76

Source: *ISSP Health and Health Care II (2021–2024) and external data sources*. Authors' calculations. (N = 35,592; 32 countries). Note. Includes all statistically relevant PCA-derived indices simultaneously. *** P < .001, ** P < .01, * P < .05

INTERACTIONS

Table S12. Cross-level interaction models

	M11a			M11b		
**Variables**	**Logit**	**r. SE**	**OR (95% CI)**	**Logit**	**r. SE**	**OR (95% CI)**
Fieldwork year	0.10	0.06	1.11 (0.99, 1.25)	0.11	0.06	1.12 (1.00, 1.26)
Gender (*female*)	0.36***	0.05	1.44 (1.31, 1.58)	0.32***	0.06	1.38 (1.23, 1.56)
Age (*cohorts*)	-0.42***	0.06	0.65 (0.58, 0.73)	-0.42***	0.06	0.65 (0.58, 0.73)
Education (*degree levels*)	0.38***	0.04	1.46 (1.36, 1.57)	0.38***	0.04	1.46 (1.36, 1.56)
Employment status *(active)*	0.28*	0.11	1.31 (1.05, 1.64)	0.28*	0.11	1.33 (1.06, 1.66)
Partnership *(partner)*	0.29***	0.06	1.34 (1.20, 1.50)	0.29***	0.06	1.33 (1.19, 1.49)
Health problems	0.48***	0.09	1.62 (1.34, 1.96)	0.48***	0.09	1.61 (1.34, 1.95)
Smoking	-0.002	0.02	1.00 (0.96, 1.03)	0.000	0.02	1.00 (0.97, 1.04)
Alcohol use	0.03	0.02	1.03 (0.98, 1.08)	0.03	0.02	1.03 (0.99, 1.08)
Physical activity	0.09**	0.04	1.10 (1.02, 1.18)	0.09**	0.04	1.10 (1.02, 1.18)
Disability	-0.08	0.09	0.92 (0.78, 1.10)	-0.08	0.09	0.92 (0.78, 1.10)
Subjective health	0.08**	0.03	1.09 (1.02, 1.15)	0.08**	0.03	1.09 (1.02, 1.15)
Mental wellbeing	-0.16***	0.05	0.86 (0.78, 0.94)	-0.16***	0.05	0.85 (0.78, 0.94)
Doctor visits	0.14***	0.04	1.15 (1.06, 1.24)	0.14***	0.04	1.15 (1.06, 1.24)
Satisfaction with doctor visits	-0.06	0.03	0.94 (0.88, 1.00)	-0.05	0.03	0.95 (0.89, 1.01)
No doctor visit	-0.97**	0.31	0.38 (0.21, 0.69)	-0.94***	0.31	0.39 (0.22, 0.71)
Unmet healthcare needs	0.33**	0.11	1.39 (1.13, 1.72)	0.33**	0.11	1.39 (1.13, 1.72)
Not need medical treatment	-0.08	0.06	0.92 (0.82, 1.03)	-0.07	0.06	0.93 (0.83, 1.04)
Trust in doctors	-0.13***	0.03	0.88 (0.83, 0.94)	-0.12***	0.03	0.88 (0.83, 0.95)
Confidence in healthcare	0.04	0.05	1.05 (0.96, 1.14)	0.04	0.05	1.04 (0.96, 1.14)
Satisfaction with healthcare	-0.02	0.04	0.98 (0.91, 1.07)	-0.02	0.04	0.99 (0.91, 1.07)
Perceived quality of care	0.001	0.04	1.00 (0.93, 1.08)	0.005	0.04	1.01 (0.93, 1.09)
Perceived access to care	0.03	0.06	1.03 (0.90, 1.17)	0.02	0.06	1.02 (0.90, 1.16)
Perceived usefulness of I	0.44***	0.04	1.56 (1.45, 1.68)	0.47***	0.04	1.60 (1.49, 1.71)
Unreliability of information	0.07*	0.03	1.07 (1.01, 1.13)	0.08*	0.02	1.08 (1.03, 1.14)
Country-level variables						
Development	0.62***	0.11	1.85 (1.49, 2.32)	0.68***	0.10	1.97 (1.61, 2.42)
Interaction effect						
Development ## gender	0.16**	0.06	1.17 (1.03, 1.31)			
Development ## perceived usefulness of internet				0.09***	0.02	1.09 (1.05, 1.14)
Others						
Constant	1.70	0.17		1.74	0.18	
Country variability	0.25	0.07		0.26	0.07	
ICC			0.07			0.07
AIC			20463.29			20462.56
BIC			20709.2			20708.48
BIC diff			-10.1			-10.9
-2LL			-10202.64			-10202.28

Source: *ISSP Health and Health Care II (2021–2024) and external data sources.* Authors' calculations. (N = 35,592; 32 countries). Note. Models include interaction terms between selected individual-level variables and country-level indices. *** P < .001, ** P < .01, * P < .05.
